# Molecular Characteristics of Radon Associated Lung Cancer Highlights MET Alterations

**DOI:** 10.3390/cancers14205113

**Published:** 2022-10-19

**Authors:** Gabriele Gamerith, Marcel Kloppenburg, Finn Mildner, Arno Amann, Sabine Merkelbach-Bruse, Carina Heydt, Janna Siemanowski, Reinhard Buettner, Michael Fiegl, Claudia Manzl, Georg Pall

**Affiliations:** 1 Department of Haematology and Oncology, Clinic of Internal Medicine V, Medical University of Innsbruck, 6020 Innsbruck, Austria; 2 Clinic of Otorhinolaryngology—Head & Neck Surgery, Medical University of Innsbruck, 6020 Innsbruck, Austria; 3 Institute of Pathology, University Hospital Cologne, 50937 Cologne, Germany; 4 Clinic Hochrum, 6063 Rum, Austria; 5 Institute of Pathology, Neuropathology and Molecularpathology, Medical University of Innsbruck, 6020 Innsbruck, Austria

**Keywords:** radon exposure, lung cancer, genetic profile

## Abstract

**Simple Summary:**

Lung cancer (LC) is the leading cause of cancer death worldwide. After smoking, one of the most prominent risk factors for LC development is radon (Rn) exposure. In our study we analysed and compared the genetic landscape of LC patients from a Rn exposed village with local matched non-exposed patients. Within the concordant genetic landscape, an increase in genetic MET proto-oncogene, receptor tyrosine kinase (MET) alteration in the Rn-exposed cohort was monitored, underlining the importance of routine MET testing and potential to enable a more effective treatment for this specific subgroup.

**Abstract:**

Effective targeted treatment strategies resulted from molecular profiling of lung cancer with distinct prevalent mutation profiles in smokers and non-smokers. Although Rn is the second most important risk factor, data for Rn-dependent driver events are limited. Therefore, a Rn-exposed cohort of lung cancer patients was screened for oncogenic drivers and their survival and genetic profiles were compared with data of the average regional population. Genetic alterations were analysed in 20 Rn-exposed and 22 histologically matched non-Rn exposed LC patients using targeted Next generation sequencing (NGS) and Fluorescence In Situ Hybridization (FISH). Sufficient material and sample quality could be obtained in 14/27 non-exposed versus 17/22 Rn-exposed LC samples. Survival was analysed in comparison to a histologically and stage-matched regional non-exposed lung cancer cohort (*n* = 51) for hypothesis generating. Median overall survivals were 83.02 months in the Rn-exposed and 38.7 months in the non-exposed lung cancer cohort (*p* = 0.22). Genetic alterations of both patient cohorts were in high concordance, except for an increase in MET alterations and a decrease in TP53 mutations in the Rn-exposed patients in this small hypothesis generating study.

## 1. Introduction

Besides the main risk factor, smoking, which is responsible for approximately 80% of LCs, another 10–25% of LC are diagnosed in never-smokers (LCINS) [1,2,3,4]. Rn exposure is the most important risk factor in never-smokers [5,6], and the second most important in LC of smokers, and is therefore associated with 9% of all deaths caused by LC and 2% of all cancer deaths in Europe [7]. Rn 222, an environmental radioactive pollutant-gas, is mainly released from the decay of uranium 238 in rock and soil causing alpha and beta emissions [7,8]. As it is electrically charged, it can attach to natural aerosol or dust and tends to be deposited on the bronchial epithelial cells. This explains its organ specific risk for lung cancer due to the cells’ exposure to this local radiation [7,9], which is well known to create molecular changes, such as DNA double-strand breaks, mutations, translocation or gene deletions [10].

Indoor Rn accumulates by structural defects in basements [11]. The correlation between high indoor Rn-exposure and LC was extensively studied in the 1990s and 2000s and a significant excess of LC due to this residential exposure was confirmed, for example, in a review of 7148 LC patients compared to 14,208 controls by Darby et al. [7]. The World Health Organization (WHO) recommends levels below 100 Bq/m^3^ [5], even though some studies observed an increase in LC risk already for 50 Bq/m^3^ [12,13]. Most find a linear risk increase of 11–16% per 100 Bq/m^3^ [7,14,15], but also a non-linear dose-response relationship has been reported [16]. Even though Rn is the most important risk factor for LCINS and the second important in smokers, few works describe molecular profiles and no detailed outcome data are available. Most available data represent uranium miners, who suffer a higher risk of LC associated with the Rn exposure in mines [17,18,19]. Nevertheless, these cohorts are biased—most miners are male smokers and many other radioactive chemicals and carcinogens, such as arsenic, silica or diesel, exist in these mines. Within those cohorts, TP53 and EGFR are the most abundantly studied genes. The largest investigation found that epidermal growth factor receptor (EGFR), tumor protein p53 (TP53), NK2 homeobox1 (NKX 2.1), phosphatase and tensin homolog (PTEN), chromodomain helicase DNA binding protein 7 (CHD7), discoidin domain receptor tyrosine kinase 2 (DDR2), lysine methyltransferase 2C (MLL3, approved abbr. is KMT2C), chromodomain helicase DNA binding protein 5 (CHD5), FAT atypical cadherin 1 (FAT1) and serine/threonine/tyrosine interacting like 2 (DUSP27, now: STYXL2), LAK receptor tyrosine kinase (ALK), ret proto-oncogene (RET), AKT serine/threonine kinase 1 (AKT1), B-Raf proto-oncogene, serine/threonine kinase (BRAF), catenin beta 1(CTNNB1), erb-b2 receptor tyrosine kinase 2 (ERBB2), KRAS proto-oncogene, GTPase (KRAS), mitogen-activated protein kinase kinase 1 (MAP2K1), MET proto-oncogene, receptor tyrosine kinase (MET), NRAS proto-oncogene, GTPase (NRAS), phosphatidylinositol-4,5-bisphosphate 3-kinase catalytic subunit alpha (PIK3CA) out of 37 cancer susceptive genes related to LCINS were associated with Rn exposure [20]. The results of those molecular investigations are summarized in Table 1 [7,19,20,21,22,23,24,25].

Therefore, we investigated a local cohort with high Rn-exposure for survival and genetic alterations of oncogenic drivers in comparison to a regional non-exposed LC cohort.

## 2. Materials and Methods

### 2.1. Patients and Patient Material

This study was approved by the regional ethical board (AN 1018/2018). Histologically confirmed, adult LC patients from 1995 to 2009 with resident addresses in Umhausen, the Rn-exposed region, were included and their Rn-exposure was assessed based on results from a previous study on longitudinal Rn-exposure in this region. In total 20 patients were identified and outcomes, characteristics and treatments, as well as risk factors of the patients were evaluated based on the documented patient’s histories. For 2/20, a second lung tumour sample was available and analysed, accounting for analyses of 22 samples from 20 patients. To identify the non-exposed LC cohort the TYROL registry of LC patients resident in Tyrol and the FUZZY matching tool of IBM SPSS Statistics were used. We matched the patients based on their age at diagnosis and gender. In addition, for molecular testing, histology and year of diagnosis was most important, whereas for survival we added the Union for International Cancer control stage based on clinical tumour assessment (cUICC). Smoking history could not be adequately matched based on missing information in this historical cohort.

### 2.2. Molecular Analyses

#### 2.2.1. Fluorescence In Situ Hybridization (FISH)

In order to detect gene fusions or amplifications of specific oncogenic drivers, FISH analyses on formalin-fixed paraffin-embedded tumour tissue (FFPE) were performed. In brief, 1–3 µm tumour sections were used, and after tissue pre-treatment nuclear DNA and the respective probes were denaturated for 5 min at 80 °C followed by a hybridization step at 37 °C for 18 h. The following probes were used: ALK (ALK Dual Color Break apart Probe, Zytovision, Bremerhaven, Germany), RET (RET Dual Colour Break apart Probe, Zytovision), ROS1 (ROS1 Dual Colour Break apart Probe, Zytovision) and MET (MET/CEN17 probe, Vysis, Downers Grove, IL, USA). Tissue was mounted in a DAPI containing mounting media (Zytovision) and interphase nuclei were monitored using a ZEISS axioplan2 microscope equipped with a 63× oil objective (Zeiss, Oberkochen, Germany) and a Progress GRYPHAX SUBRA camera/software (Jenoptik, Jena, Germany). Evaluation was performed as previously described [26,27,28,29].

#### 2.2.2. Parallel Sequencing (Next Generation Sequencing, NGS)

For DNA extraction three to six 10 µm-thick sections were cut from FFPE tissue. Sections were deparaffinized and the tumour areas were macro-dissected from unstained slides using a marked haematoxylin-eosin (H&E) stained slide as a reference. After proteinase K digestion, the DNA was isolated with the Maxwell^®^ 16 FFPE Plus Tissue LEV DNA Purification Kit (Promega, Mannheim, Germany) on the Maxwell^®^ 16 (Promega) following manufacturer’s instructions. For Next Generation Sequencing, the DNA concentration and integrity was measured using a quantitative real-time PCR (qPCR). Multiplex PCR-based parallel sequencing was performed on all FFPE samples (Table S1). Isolated DNA was amplified with an Ion AmpliSeq Custom DNA Panel (Thermo Fisher Scientific, Waltham, MA, USA) and the Ion AmpliSeq Library Kit 2.0 (Thermo Fisher Scientific) following manufacturer’s instructions. The gene panel comprised relevant exons of the following genes: AKT1, ALK, BRAF, CTNNB1, DDR2, EGFR, ERBB2, KRAS, MAP2K1, MET, NRAS, PIK3CA, PTEN and TP53 (Table S2: detailed investigated exons and HGNC full names of genes). After end-repair and adenylation, NEXTflex DNA Barcodes were ligated (Bio Scientific, Austin, TX, USA). Barcoded libraries were amplified, final library products were quantified, diluted and pooled in equal amounts. Finally, 12 pM of the constructed libraries were sequenced on the MiSeq (Illumina, San Diego, CA, USA) with a MiSeq reagent kit V2 (300-cycles) (Illumina) following manufacturer’s recommendations.

#### 2.2.3. Measurement of Indoor Radon Levels

All Rn measurements were performed within the study “Radon und Lungenkrebs im Bezirk Imst/Tirol” by W. Oberaigner et al. [30] in 2002, where long-term measurements in basements and ground floors indoors were used. Ennemoser et al. measured concentrations with medians ranging from 361–3750 Bq/m^3^ (basements) and 210–1.160 Bq/m^3^ (ground floors) in summer and winter, respectively [31]. The maximum Rn concentration which was measured was 274.000 Bq/m^3^ [31].

### 2.3. Data/Statistical Analysis

Data were analysed with IBM SPSS Statistics 24 (IBM Corp., Armonk, NY, USA). For survival analyses Kaplan-Meier plots and log-rank tests were performed.

NGS data were exported as FASTQ files. Alignment and annotation were done using a modified version of a previously described method [32]. BAM files were visualized in the Integrative Genomics Viewer (http://www.broadinstitute.org/igv/ (accessed on 1 June 2021), Cambridge, MA, USA). A 5% cut-off for variant calls was used and results were only interpreted if the coverage was >200×.

## 3. Results

### 3.1. Patients Characteristics

Patient characteristics are given in detail in Table 2. For the Rn-exposed patients we were able to match 20 patients for molecular testing based on histology and year of diagnosis besides age and gender. In the overall non-exposed LC cohort used for the survival analyses, 52 patients based on cUICC were matched including those for molecular testing.

In total, the mean age of patients at time of diagnosis was 63.0 in the Rn-exposed and 63.4 years in in the non-exposed LC cohort with a gender distribution of 9f:11m and 21f:31m, respectively. According to cUICC stages (at diagnosis) the Rn-exposed cohort consisted of three patients in stage I (20%), three in stage II (20%), four in stage III (26.7%) and five in stage IV (33.3%). The stage of the remaining patients (*n* = 5) was not available. The non-exposed LC cohort comprised 12 patients in stage I (23.1%), 9 in stage II (17.3%), 17 in stage III (32.7%) and 14 in stage IV (26.9%). ECOG data were not available for all patients; however, the distribution was similar between the two groups, with 76.9 and 86.7% with ECOG 0–1, 7.7 and 11.1% with ECOG 2. Single patients had an ECOG > 2, two in the Rn-exposed cohort and one in the non-exposed LC cohort, respectively. 40% of Rn-exposed tumours were classified as adeno-, 50% as squamous cell carcinomas. In the overall non-exposed LC cohort, we had a slight bias towards adenocarcinomas in comparison to the Rn-exposed patients 52.0 vs. 40.0%, respectively, and 42.3 percent were squamous carcinomas, besides singular rare histological subtypes (Table 2). Smoking history of analysed patients was not comparable between the two cohorts, as information for half of the patients in the Rn-exposed group was missing. However, the percentage of patients with proven/evidenced smoking history in the non-exposed LC cohort (77.2%) was numerically higher than in the Rn-exposed group (60%).

### 3.2. Molecular Profile

**Figure 1:**
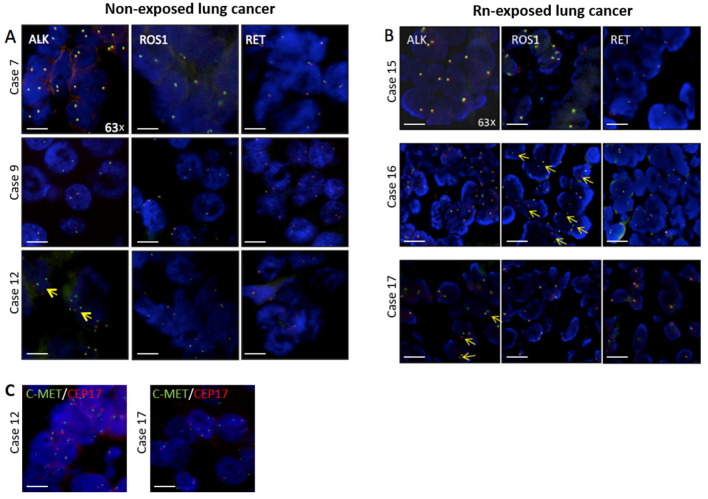
Fluorescence in-situ hybridization images showing ALK, ROS1 and RET signals of representative cases of (**A**) non-exposed LC group (*n* = 3) and (**B**) Rn-exposed group (*n* = 3). Yellow arrows highlight split signals (1F 1O 1G; insertion) or single red (in the case of ALK and ROS break apart probes) and single green signals (RET probe), both indicating insertion with deletion. (**C**) FISH images showing c-MET signal (green) and CEP17 signals (red) in two exemplary non-amplified cases. Images were taken using an Axioplan 2 (Zeis) microscope equipped with a 63× oil objective and a progress GRYPHAX SUBRA camera/software (Jenoptik). Scale bar = 50 µm.

In order to monitor genetic alterations in the two patient cohorts, an NGS based testing for AKT1, ALK, BRAF, CTNNB1, DDR2, EGFR, ERBB2, KRAS, MAP2K1, MET, NRAS, PIK3CA, PTEN and TP53 genes was performed and translocations of ALK, ROS and RET and amplification of MET were analysed by FISH (Figure 1; Table 3).

Sufficient material and sample quality could be obtained in 14/27 non-exposed versus 17/22 Rn-exposed LC samples for NGS read out and FISH analyses, respectively (see Table 3). Additionally, some samples were feasible for one method, but not for the other technique. This might be due to low DNA quality and/or quantity or an under- or over-fixation with formalin of the tissue. In two Rn-exposed patients two samples from different time points within the course of their disease were analysed—the first case was a squamous cell carcinoma without alteration at both time points. A second lung cancer primary was detected in one patient 6.5 years after the first diagnosis including a switch in histology from squamous cell carcinoma without any alterations to an adenocarcinoma with a MET mutation.

**Figure 2:**
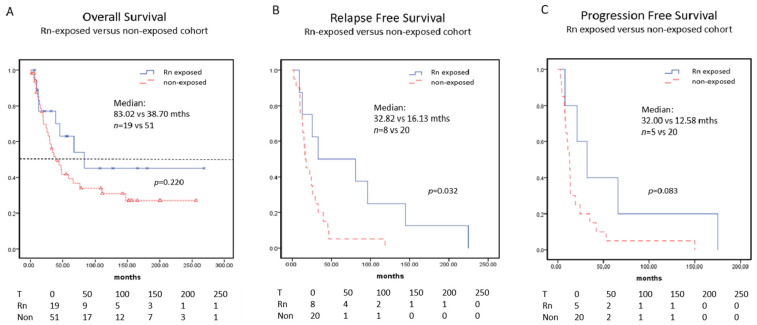
Kaplan–Meier survival analysis-non-exposed (red dotted line) versus Rn-exposed subgroup (blue line). (**A**) overall survival (*n* = 51 vs. 19; median survival of 38.7 vs. 83.0 mths), (**B**) relapse free survival (*n* = 20 vs. 8; median survival of 16.13 vs. 32.82 mths) and (**C**) progression-free survival (*n* = 20 vs. 5; with median survival of 12.85 vs. 32 mths) of non-exposed and Rn-exposed groups.

In general, a higher number of patients with genetic alterations of these known driver genes were detected in the non-exposed LC cohort compared to the Rn-exposed cohort (Figure 2, Table 3), but the latter had more different alterations. Genetic alterations in PTEN and ERBB2 genes were each found in one patient of the non-exposed LC cohort. The patient showing an activating exon 20 ERBB2 alteration (c.2332_2340dup; p.G778_P780dup) also showed a deletion in TP53 (c.276–1_391del), with an unknown consequence for protein function. The case with PTEN mutation showed two different alterations in this gene, i.e., c.274G>C (p.D92H), resulting in a loss of function and c.860C>G (p.S287), with a potential loss of function of PTEN.

Mutations in the oncogene KRAS were determined in 1/14 (7%) and 2/17 (12%) in the non-exposed and Rn-exposed cohort, respectively. All detected mutations were in codon 12 (exon 2), representing a base exchange leading to G12C, V or D. Exclusive genetic alterations in the tumour suppressor gene TP53 were monitored in 8/14 (57%) and 3/17 (18%) patients of the non-exposed and Rn-exposed cohorts, respectively. In the Rn-exposed cohort less TP53 mutations were depicted while a higher number of KRAS mutations and MET alterations were detected compared to the non-exposed LC cohort. One case of the non-exposed LC cohort showed a duplication in ERBB2 gene in parallel and in one patient of the Rn-exposed cohort a concomitant variation of MET, c.3029C>T; p.T1010I was found. Interestingly, another case in the Rn-exposed cohort expressed the same T1010I mutation in the MET gene and also in this case it was a concomitant alteration. In this tumour, with the histological subtype of adenocarcinoma, an additional translocation in the anaplastic lymphoma kinase (ALK) could be monitored by FISH analysis, the only one in the Rn-exposed cohort (1/17; 6%). In comparison in the non-exposed LC cohort, 2/14 (14%) harboured an ALK translocation. Mutations in the MET gene were only detected in theRn-exposed group, 2/17 (6%) as concomitant alterations (p.T1010I) and 2/17 (6%) as sole mutation/insertion-deletion (c.3082 + 3A>T; c.2942–15_2942–4delinsACACA) of the juxtamembrane domain, but all four mutations were located in exon 14 of MET. Additionally, in one tumour sample of the Rn-exposed cohort an amplification in MET was determined with FISH analysis (6%). Testing for further translocations (fusion proteins) in ROS1 and RET one ROS1 positive case in the Rn-exposed group could be monitored, while no positive result for RET translocation was found.

Summarizing, investigation of this hotspot panel of genetic alterations resulted in (i) a higher number of non-mutated patients in the Rn-exposed cohort compared to the non-exposed group (32 vs. 7%), with (ii) an increased number of TP53 alterations in the non-exposed LC cohort and (iii) an unexpectedly high amount of MET alterations solely in the Rn-exposed patient cohort (Table 3).

### 3.3. Number of Mutations in Correlation with Level of Rn-Exposure

The Rn exposure-levels of 12/20 patients of our Rn-exposed cohort varied between 209 Bq/m^3^ and 29,970 Bq/m^3^. Due to the limited available data concerning the Rn exposure (63%), i.e., doses and time, no correlation of number and kind of genetic alteration and radon exposure could be assessed.

### 3.4. Survival

As shown in Figure 2 the Rn-exposed group had a longer median overall survival (mOS) with 83 months compared to the non-exposed group with a mOS of 39 months, but this difference did not reach statistical significance due to the low patient numbers (*p* = 0.22). Similarly, relapse free survival was longer in the Rn-exposed cohort (median 81 vs. 33 months, *p* = 0.84). Stage dependent survivals are given in Supplementary Table S3.

**Figure 3:**
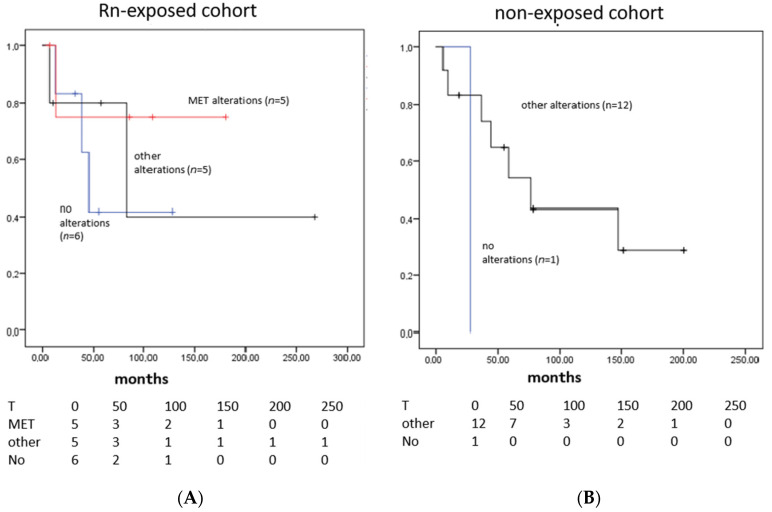
Overall survival analysis stratified by summarized genetic alterations in patients of (**A**) Rn-exposed LC cohort and (**B**) non-exposed LC cohort. The red line represents MET alterations, and the black line, all other alterations (mainly TP53 and KRAS); the blue line depicts those without alterations.

Stratification of survival data in different genetic sub-groups reflects well-known characteristics with short overall survivals in historical cohorts and worse survivals for a the mainly TP53 and KRAS mutated subgroup or a sub-cohort with no specific drivers tested, whereas MET mutated or amplified patients displayed longer survivals (see Figure 3).

Statistical analyses were not performed due to low patient numbers and the results need to be interpreted with caution based on the limitations in patient numbers. Nevertheless, these findings warrant further analyses of survivals in Rn-exposed patients and especially in MET mutated cohorts, even if no conclusions can be drawn from our study.

## 4. Discussion

Rn is the most important risk factor for LC in never smokers and the second in smokers [5,6], but the knowledge about specific mutations or mutation-frequencies in potentially Rn-related NSCLC remains low and no outcome data on Rn-related lung cancer patients are available so far. The best-investigated gene is TP53, but none of the Rn-specific mutations that were detected in previous studies could be validated by others [17,18,19,21,22]. In this study we screened for alterations in ALK, AKT1, BRAF, CTNNB1, DDR2, EGFR, ERBB2, KRAS, MAP2K1, MET, NRAS, PIK3CA, RET, ROS1, PTEN and TP53 genes in Rn-exposed NSCLC patients from a village in Tyrol, where high Rn exposure due to the soil texture occurred.

Rn exposures measured for our cohort with >300 Bq/m^3^ in 81.8% of the available cases were higher than in other studies [20,23,33] and far exceed the WHO recommended limit of 100 Bq/m^3^ [5]. This emphasizes Umhausen as an area with extensive environmental Rn exposure.

Our Rn-exposed cohort presented with a median survival of 83 months compared to our local matched non-exposed group with 39 months. This result is limited by the low numbers and retrospective character of this analysis with lack of statistical significance. Some clinical data in this retrospective series were missing, including ECOG status and smoking habits in several cases. A higher awareness of cancer risk might contribute to early detections and better survivals, but clinical data and stages for the survival analyses were matched between the cohorts and patient characteristics were well balanced. Hence, a genetic background seems reasonable. On the one hand, in the Rn-exposed LC patients less TP53 mutations were detected, which are known negative prognostic factors and might translate into better survival [34]. In addition, TP53 mutations are linked to platin resistance, which was one of the only available standard drugs at the time of patient treatment courses [35]. In addition, radiation causing a higher rate of DNA damage might overwhelm functioning repair mechanisms including TP53, which might also explain tumorgenesis even in TP53 wt individuals and supports a different genetic background. On the other hand, MET mutations were increased, but their specific clinical prognostic relevance remains to be determined. Of note, at time of diagnosis and initial treatment no targeted drugs were available, and hence were not applied or were used very late in the course of the disease. Furthermore, several other metabolic, genomic and clinical patterns were not assessed. Nevertheless, to our knowledge, this is the first study to show data concerning survivals and genetic mutations in Rn-exposed LC subgroups, which hint towards a distinct genetic background and therapeutic responsiveness. This should encourage future studies of this specific patient population.

Concerning the molecular alterations, TP53 mutations were less common in our Rn-exposed cohort (20%) in comparison to NSCLC patients of our non-exposed group (64%) and in the AACR project (50.3%) [36]. Our results are in line with the findings of a large review of Ruano-Ravina et al. including 578 individuals reporting a frequency of TP53 mutations in 26% in uranium miners and 24% in environmental Rn-exposed patients [22], and with Choi et al. in a Rn-exposed Asian cohort (21%) [20]. In summary, Rn-exposed people showed less TP53 mutations and neither we nor others found specific Rn-induced mutations.

KRAS mutations occurred in 10% of the Rn-exposed cohort and in 7% in the non-exposed cohort; both percentages are below pre-described frequencies of 29.7% of NSCLC-patients in the AACR project [36]. McDonald et al. described KRAS mutations as more frequent in Rn-exposed uranium miners [37]. Choi et al. described KRAS mutations in 5.0% of their Rn-exposed study population [20]. The limited numbers hamper our results, but differences might well be due to differences in the investigated cohorts. Uranium miners are exposed to several carcinogens and most of them were smokers and had adenocarcinomas, which harbour more KRAS mutations than found in squamous cell carcinoma in lung [36]. This is even more interesting, as both our cases occurred in squamous cell carcinomas. The G12V and G12C mutations in our patients are well known and frequent mutations in LC and colorectal adenocarcinoma [36,38]. The mutations are associated with a worse prognosis [39] but are unlikely to have been caused exclusively by Rn-exposure. Recent developments offer a targeted drug for this cohort, which encourages standard testing [40]. For re-arrangements in ALK, ROS1 and RET neither higher incidence, nor Rn-specificity was found. Only one, the ROS1 + case, received a documented targeted treatment with crizotinib in 3rd line, due to diagnoses prior to any targeted treatment options.

The analyses of MET gene alterations showed the most interesting result. While in the non-exposed lung cancer cohort no tumour had an alteration in the MET gene, five patients (25%) of the Rn-exposed cohort showed alterations in this gene, whereof four tumours showed a substitution and missense mutation (for detail see Table S1) and in one tumour there was an amplification of MET, although classified as low level. In addition, the results of the AACR-Project, identifying 5.8% of patients with MET mutations and 0.1% with MET amplifications [36], underlines the low frequency of MET alterations in lung cancers. However, the T1010I variation that we found in two cases is very rare. Of note, this T1010I MET mutation is a reported SNP (rs56391007) with 1.2% in a European sub-population [41]; however, the clinical significance is still under discussion and reported data are conflicting. A correlation to Rn-associated tumours remains to be clarified in a bigger study. This specific mutation was additionally described as relatively frequent in differentiated thyroid carcinoma [42], and an increase of growth factor independent proliferation and motility in in-vitro tumour cell lines with a T1010I mutation [43] were described. In contrast, Voortman et al. described the T1010I mutation as not resulting in enhanced Met phosphorylation [44], which questions its role in oncogenesis [44]. Both patients in the Rn-exposed cohort with T1010I had an additional genetic alteration in another investigated gene: one tumour had an ALK translocation and one tumour a mutation in TP53. This could diminish the relevance, at least of the T1010I mutation, as an oncogenic driver and therefore the importance remains elusive. So far, only one study conducted by Choi et al. investigated MET mutations in Rn-exposed people. They described an alteration frequency of 5% in MET in this cohort of 19 LC patients. All four MET alterations occurred in Exon-14, and Exon-14 skipping mutations are a well-known driver of alteration in LC. Therefore, our findings might be clinically relevant, as capmatinib [45], tepotinib [46] and savolitinib [47], are specific MET-inhibitors. Quite recently, the FDA and/or EMA approved therapeutics for MET-Exon-14 skipping alterations in NSCLC, and crizotinib [48,49], a tyrosine kinase inhibitor (TKI) targeting ALK, MET and ROS is under clinical development with inclusion criteria of MET-mutations.

Of note, for the T1010I mutation bearing and MET amplified patients the smoking status was unknown, but both other MET mutations occurred in former smokers, who quit smoking at least two years prior to diagnosis.

Several limitations apply to our analysis. The limited number of patients precludes definitive conclusions and renders the results hypothesis generating. Furthermore, the retrospective data collection, with, at least in part, missing information on important prognostic factors (i.e., performance-status, smoking-history), might have confounded the survival-outcome.

Hence, our results encourage further studies in a larger cohort of Rn-exposed LC patients to highlight the potential correlation between Rn exposure and MET mutations as well as KRAS mutations and inclusion of Rn-exposed LC patients within the screening for trials investigating MET and KRAS inhibitors.

## 5. Conclusions

Within a Tyrolean NSCLC cohort exposed to high environmental Rn levels (81.8% >300 Bq/m^3^) a long median survival of 83 months was observed. This might be due to less TP53 mutations compared to reported numbers and a local control group, as well as an increased number of genetic alterations in the MET-gene, even though results are limited by the small patient numbers and the retrospective character of this study. Nevertheless, alterations in the MET gene in general and the T1010I mutation specifically are very rare in LC patients and hence an association with Rn-exposure seems reasonable.

## Figures and Tables

**Table 1:** Overview of previous studies.

Author (Ref.)	Year	Region	Subjects	Gene
S. Darby et al. [7]	2005	Europa	7142 patients + 14,208 controls	-
K. Vahakangas et al. [17]	1992	Europa	19 uranium miners	TP53
J.A. Taylor et al. [19]	1994	America	52 uranium miners	TP53
Q. Yang et al. [21]	2000	Germany	79 uranium miners	TP53
A. Ruano-Ravina et al. [22]	2009	Worldwide	578 individuals	TP53
A. Ruano-Ravina et al. [23]	2016	Spain	323 Patients	EGFR/ALK
M. Taga et al. [24]	2012	USA	70 women	EGFR
Bonner et al. [25]	2006	USA	270 individuals	GSTM1
J.R. Choi et al. [20]	2017	Asia	19 patients	EGFR/TP53/NKX 2.1/PTEN/CHD7/DDR2/MLL3/CHD5/FAT1/DUSP27

**Table 2:** Patients’ characteristics.

	Rn-Exposed LC Cohort *n* = 20	Non-Exposed LC Cohort *n* = 52
Mean age at diagnosis	63.0 years	63.4 years
Gender (female/male)	9/11	21/31
Alive/dead	12/8	20/31 *
UICC		
I	3 (20.0%)	12 (23.1%)
II	3 (20.0%)	9 (17.3%)
III	4 (26.7%)	17 (32.7%)
IV	5 (33.3%) *	14 (26.9%)
ECOG		
0–1	10 (76.9%)	39 (86.7%)
2	1 (7.7%)	5 (11.1%)
>2	2 (15.4%) *	1 (2.2%) *
Histology		
Adenocarcinoma	8 (40.0%)	27 (52.0%)
Squamous cell carcinoma	10 (50.0%)	22 (42.3%)
Adenosquamous cell carcinoma	1 (5.0%)	0 (0.0%)
Large cell carcinoma	0 (0.0%)	2 (3.8%)
Large cell neuroendocrine carcinoma	1 (5.0%)	0 (0.0%)
NOS	0 (0.0%)	1 (1.9%)
Smoking		
Smoker or former smoker	6 (30.0%)	34 (65.5%)
Never smoker	4 (20.0%)	10 (19.2%)
Unknown	10 (50.0%)	8 (15.3%)

* information for some patients is missing. ECOG = Eastern Cooperative Oncology Group.

**Table 3:** Basic overview of genetic alterations.

Cohort		Frequency (*n*)	Percent (%)
Non-exposed	no alterations	1	7
	KRAS_mut	1	7
	TP53_mut	8	57
	TP53/ERBB2mut	1	7
	PTEN mut	1	7
	ALK-rearr	2	14
Rn-exposed	no alterations	6	35
	KRAS_mut	2	12
	TP53_mut	3	18
	ROS-rearr	1	6
	MET-amplification	1	6
	MET_mutation	2	12
	MET T1010I, ALK rearr	1	6
	MET T1010I, TP53_mut	1	6

## Data Availability

The data presented in this study are available on request from the corresponding author. The data are not publicly available due to ethical restrictions.

## Supplementary Materials

The following supporting information can be downloaded at: https://www.mdpi.com/article/10.3390/cancers14205113/s1, Table S1: Primer for targeted NGS; Table S2: Specific genes and exons for targeted NGS panel: NGS_LUN3_#3; Table S3: Median overall survivals in months of the radon exposed, matched non-exposed cohort.
