# Supplementary material for: Molecular Characteristics of Radon Associated Lung Cancer Highlights MET Alterations
Source: Cancers (Basel). 2022 Oct 19;14(20):5113. doi: 10.3390/cancers14205113 (PMC9600309; doi:10.3390/cancers14205113)
Supplement: Supplementary file 1 [file cancers-14-05113-s001.zip › cancers-1945801-supplementary.pdf]

Supplementary tables

Table S1: Primer for targeted NGS

Chrom.	Start	End	F-Primer	R-Primer		Gen
chr2	29432650	29432776	GGGTGAGGCAGTCTTTACTCA	CGTTGTACACTCATCTTCCTAGGGAT	AMPL403782807	ALK Exon25
chr2	29436843	29436974	ACAGGAAGAGCACAGTCACTTTG	CTCACTGACAAGCTCCTCGT	AMPL404264901	ALK Exon24
chr2	29443565	29443688	ACTGCAGCAAAGACTGGTTCTC	GCTCTGCAGCAAATTCAACCAC	AMPL403021863	ALK Exon23
chr2	29443688	29443772	CCAATGCAGCGAACAATGTTCTG	AGCTGCTGAAAATGTAACTTTGTATCCT	AMPL403042136	ALK Exon23
chr2	29445200	29445332	GGGTGAGGGTGTCTCTCTG	TTGGCTTGCGGACTCTGTAG	AMPL403100866	ALK Exon22
chr2	29445369	29445489	AAGGGCAGGCTCAAGAGTG	CTGTTTGACTCTGTCTCCTCTTGT	AMPL401739236	ALK Exon21
chr7	116411701	116411801	CTGTGGCTGAAAAAGAGAAAGCAA	CAGAACTCATGGGTTTTATGGACTACAT	AMPL3669435999	cMET Intron 13/14
chr7	116411801	116411909	CGTCGATTCTTGTGTGCTGTCTTAT	GCATCGTAGCGAACTAATTCACTG	AMPL3669440693	cMET Exon14,cMET Intron13/14
chr7	116411894	116411998	CGTCTTTAACAAGCTCTTTCTTTCTCTCT	CGGTAGTCTACAGATTCATTTGAAACCA	AMPL3669509335	cMET Intron13/14,cMET Exon14
chr7	116411998	116412072	CGAAGTGTAAGCCCAACTACAGAAA	ACAACCCACTGAGGTATATGTATAGGT	AMPL3669511201	cMET Exon14
chr12	25380167	25380240	AATGTCAGCTTATTATATTCAATTTAAACCCAC	GCAATGAGGGACCAGTACATGA	AMPL387798235	KRAS Exon3B,KRAS Exon3
chr12	25380240	25380357	GAAAGCCCTCCCCAGTCC	GCACTGTAATAATCCAGACTGTGTTTC	AMPL3669413793	KRAS Exon3B,KRAS Exon3
chr12	25398183	25398304	AAAGAATGGTCCTGCACCAGTAA	AGGCCTGCTGAAAATGACTGAATATAA	AMPL388868318	KRAS Exon2
chr12	25398304	25398379	CCAGCTCCAACTACCACAAGT	CTGGTGGAGTATTTGATAGTGTATTAACCTT	AMPL637254406	KRAS Exon2
chr17	7577017	7577142	CGCTTCTTGTCCTGCTTGCTTA	TCCTATCCTGAGTAGTGGTAATCTACTG	AMPL706844244	TP53 Exon8
chr17	7577140	7577233	GCACCTCAAAGCTGTTCCGT	CAAGGGTGGTTGGGAGTAGATG	AMPL388035064	TP53 Exon8
chr17	7577392	7577509	GGGATGTGATGAGAGGTGGAT	CCATCCTCACCATCATCACACTG	AMPL388739660	TP53 Exon7
chr17	7577508	7577611	GGCTCCTGACCTGGAGTCTT	CATCTTGGGCCTGTGTTATCTCC	AMPL387805586	TP53 Exon7
chr17	7578141	7578234	GCCACTGACAACCACCCTTAA	GGAAGGAAATTTGCGTGTGGAGTA	AMPL387841166	TP53 Exon6
chr17	7578234	7578362	GTCGAAAAGTGTTTCTGTCATCCAAA	TCAGATAGCGATGGTGAGCAG	AMPL706125298	TP53 Exon6
chr17	7578310	7578425	GACCTAAGAGCAATCAGTGAGGAA	CATCTACAAGCAGTCACAGCAC	AMPL1206820909	TP53 Exon5
chr17	7578425	7578555	CGCCTCACAACCTCCGTCAT	CTCTGTCTCCTTCCTCTTCCTACA	AMPL707393388	TP53 Exon5
chr17	7579278	7579385	GGATACGGCCAGGCATTGAAG	TCATCTTCTGTCCCTTCCCAGA	AMPL705681709	TP53 Exon4
chr17	7579385	7579502	CGTAGCTGCCCTGGTAGGTTT	CTGAAGACCCAGGTCCAGATG	AMPL651471124	TP53 Exon4
chr17	7579502	7579590	CTCTGGCATTCTGGGAGCTT	ACTGCTCTTTTCACCCATCTACAG	AMPL387829477	TP53 Exon4

Table S2: specific genes and exons for targeted NGS panel: NGS_LUN3_#3

Gen	FULLNAME<br>HGNC	Exon
AKT1	AKT serine/threonine kinase 1	4
ALK	LAK receptor tyrosine kinase	21 – 25
BRAF	B-Raf proto-oncogene, serine/threonine kinase	11, 15
CTNNB1	catenin beta 1	3
DDR2	discoidin domain receptor tyrosine kinase 2	3 – 18
EGFR	Epidermal growth factor receptor	18,19, 21
EGFR	Epidermal growth factor receptor	20
ERBB2/HER2	erb-b2 receptor tyrosine kinase 2	19, 20
KRAS	KRAS proto-oncogene, GTPase	2, 3
MAP2K1	mitogen-activated protein kinase kinase 1	2
MET	MET proto-oncogene, receptor tyrosine kinase	14
NRAS	NRAS proto-oncogene, GTPase	2, 3
PIK3CA	phosphatidylinositol-4,5-bisphosphate 3-kinase catalytic subunit alpha	9, 20
PTEN	phosphatase and tensin homolog	1 – 8
TP53	tumor protein p53	5 – 8

Table S3: Median overall survivals in months of the radon exposed, matched non-exposed cohort

UICC stage	Radon exposed cohort	Matched non-exposed cohort
I	107.6 (n=3)	45.26(n=12)
II	84.47 (n=4)	58.84 (n=9)
III	39.97 (n=4)	34.56 (n=16)
IV	16.82 (n=4)	10.45 (n=13)

Stage-dependent median survivals of the radon-exposed cohort compared with the matched non-exposed cohort.
